# Two Cases of Chronic Tubular Necrosis Presenting as Fanconi Syndrome Induced by Red Yeast Rice Choleste-Help

**DOI:** 10.3390/diagnostics15131722

**Published:** 2025-07-06

**Authors:** Kanako Mita, Shunsuke Takahashi, Satoshi Yanagida, Akihiro Aoyama, Takayuki Shiraishi, Takayuki Hamada, Yumiko Nakamura, Mariko Sato, Kento Hirose, Ryo Yamamoto, Yuya Shioda, Kaori Takayanagi, Izumi Nagayama, Yuko Ono, Hajime Hasegawa, Akito Maeshima

**Affiliations:** Department of Nephrology and Hypertension, Saitama Medical Center, Saitama Medical University, Kawagoe 350-0844, Japan

**Keywords:** red yeast rice supplement, acute tubular necrosis, Fanconi syndrome, acute kidney injury, urinary biomarker, renal transporter

## Abstract

**Background and Clinical Significance:** Although dietary supplements have often been deemed safe, some have been linked to drug-induced nephropathy due to their diverse ingredients. The aim of this report is to enhance clinical awareness of a novel and emerging cause of Fanconi syndrome due to red yeast rice supplements and to contribute new histopathological and clinical data. **Case Presentation:** We report two cases of renal dysfunction and Fanconi syndrome associated with the use of red yeast rice supplements. Both patients presented with renal impairment accompanied by elevated markers of tubular injury, hypouricemia, hypokalemia, and glucosuria, consistent with Fanconi syndrome. Following the discontinuation of the red yeast rice supplement and initiation of steroid therapy, Fanconi syndrome resolved, however, moderate renal dysfunction persisted. Urinary NGAL levels improved after treatment in both cases. KIM-1 normalized in one case but remained elevated in the other. Uromodulin recovery was complete in one case and partial in the other. Renal biopsy revealed mild tubulointerstitial nephritis, with notable shedding of proximal tubular epithelial cells. Immunohistochemical analysis demonstrated reduced expression of URAT-1, Na-K ATPase, and Na-Pi IIa in some tubules. **Conclusions:** These findings suggest that renal injury induced by red yeast rice supplements is mediated by direct proximal tubular necrosis caused by a harmful substance in the supplement, resulting in persistence of tubular dysfunction.

## 1. Introduction

Dietary supplements, while widely regarded as natural and safe, have been implicated in drug-induced nephropathy due to various substances they contain. Among these, aristolochic acid nephropathy (AAN) caused by *Aristolochia* species in traditional herbal medicines is well-documented. AAN is characterized by progressive interstitial fibrosis and renal failure [1,2,3], as well as Fanconi syndrome and proximal tubular injury [4]. Similarly, nephrotoxicity has been reported in association with star fruit consumption, particularly among patients with chronic kidney disease, due to neurotoxic substances contained in the fruit that impair renal function [5]. Taken together, the potential for dietary supplements to cause nephropathy should not be underestimated. Clinicians must remain vigilant and inquire about supplement use during patient evaluations, particularly in cases of unexplained renal dysfunction.

In March 2024, a significant number of cases of health damage, including renal impairment, were reported in Japan following the consumption of supplements containing red yeast rice. Red yeast rice contains compounds that may help reduce cholesterol levels. One of these compounds, monacolin K, shares the same chemical structure as the prescription cholesterol-lowering drug lovastatin. As a result, some individuals use red yeast rice as a supplement in an attempt to lower their cholesterol.

Approximately 3000 cases of adverse health events were reported, including 212 hospitalizations and five deaths [6]. A nationwide survey of Japanese nephrologists showed that, among 192 patients who had consumed red yeast rice and visited the nephrology department, 94.1% presented with low eGFR (<60 mL/min/1.73 m^2^). In addition, elevated urinary levels of protein, β2-microglobulin (β2-MG) and N-acetyl-β-D-glucosaminidase (NAG) were observed in these patients. Laboratory findings revealed characteristics of Fanconi syndrome, including hypokalemia, hypophosphatemia, hypouricemia, glycosuria, and metabolic acidosis. Creatine kinase levels were not elevated, suggesting no rhabdomyolysis-related kidney injury. Kidney biopsies showed predominant tubulointerstitial changes, with 50% exhibiting tubulointerstitial nephritis and 32% showing tubular necrosis. Glomerular changes were less prominent. Following product discontinuation and steroid treatment, Fanconi syndrome-related parameters improved significantly, but 87% of patients still had eGFR <60 mL/min/1.73 m^2^ at the last observation, suggesting potential for longer-term renal effects [7]. Investigations conducted by researchers at the National Institute of Health Sciences in Japan, in collaboration with other experts, have identified the presence of puberulic acid and two novel compounds, Y and Z, in the affected product batches [8]. Recent studies have suggested that puberulic acid alone induces kidney toxicity [7]. However, the mechanisms through which puberulic acid causes kidney damage remain unclear and are currently under investigation.

We report two cases of renal impairment associated with red yeast rice supplementation. Renal biopsies revealed findings of tubular necrosis. In addition, immunohistochemical staining demonstrated reduced expression of several transporters in certain tubules. Biomarkers of acute kidney injury (AKI) showed no improvement even after one month of steroid therapy. These observations suggest that the causative substance may accumulate in the renal tubules, leading to chronic tubular necrosis.

## 2. Case Reports

### 2.1. Case 1

**Figure 1:**
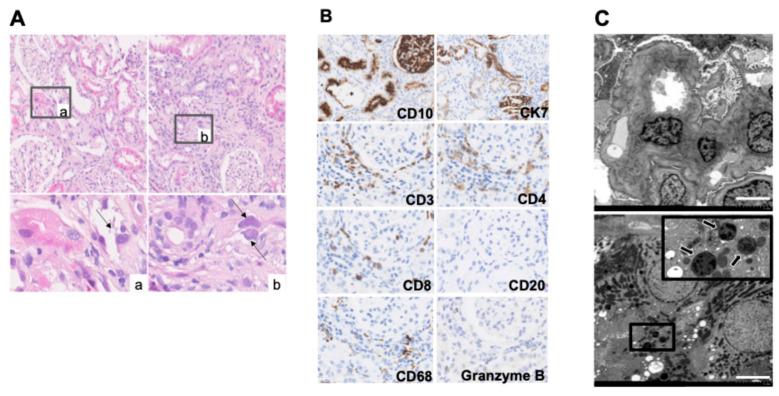
Kidney biopsy sample in Case 1. (**A**) Hematoxylin and eosin staining, magnification ×400. Large, denatured nuclei are observed (**a**,**b**: arrow). (**B**) Immunohistochemistry, magnification ×400. Tubular epithelial desquamation and tubular dilation are observed (*). CD10: proximal tubule; CK7: distal tubule; CD3, CD4, CD8: T cell; CD20: B cell; CD68: macrophage; granzyme B: cytotoxic T cell. (**C**): electron microscopy. Scale bars are 50 μm. Arrows indicate lysosomes.

**Figure 2:**
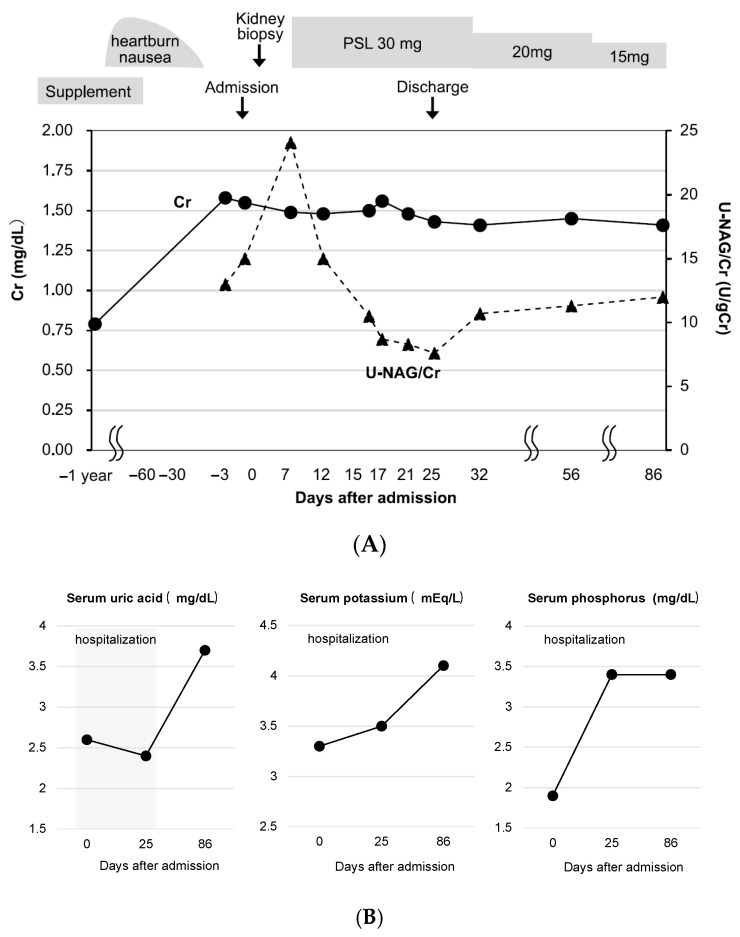
Clinical course in Case 1. (**A**) Clinical course. PSL: prednisolone. (**B**) Changes in serum levels of uric acid, potassium, and phosphorus.

A 45-year-old man presented with kidney dysfunction. His serum creatinine (Cr) level had been 0.79 mg/dL during a health checkup conducted one year earlier. He had a history of dyslipidemia diagnosed 10 years prior and no other medical history. Although no medication had been prescribed, the patient began taking red yeast rice supplements two months prior to admission. Shortly thereafter, he experienced heartburn and nausea, leading him to visit a local clinic. The supplements were discontinued and esomeprazole was prescribed. After viewing a television report linking red yeast rice supplements to kidney damage, the patient became concerned and revisited the clinic, where a urine test revealed significant proteinuria (3+). He was subsequently referred to our department for further evaluation. On admission, the patient’s height and weight were recorded as 166 cm and 45.9 kg, respectively. Physical examination revealed a body temperature of 37.6 °C and blood pressure of 153/89 mmHg. No significant physical findings, such as rashes or joint pain, were observed. Laboratory tests revealed kidney dysfunction (serum Cr, 1.78 mg/dL; eGFR, 39.7 mL/min/1.73 m^2^) and mild anemia (hemoglobin 12.1 g/dL). Urinalysis showed proteinuria (urinary protein 0.40 g/g Cr) and elevated tubular injury markers (urinary NAG, 17.0 U/L; β2-microglobulin, 6566 μg/L). Other findings included hypouricemia (uric acid, 3.4 mg/dL), hypokalemia (potassium, 3.5 mEq/L), decreased tubular phosphate reabsorption (%TRP, % tubular reabsorption of phosphate, 74.6%), and glycosuria (urine glucose 3+), consistent with Fanconi syndrome. Abdominal computed tomography (CT) showed no renal atrophy, and gallium scintigraphy revealed no gallium uptake in the kidneys. Kidney biopsy was performed on day 2. Light microscopy revealed a mild increase in mesangial matrix without glomerulosclerosis. Tubular injury was noted, predominantly affecting the proximal tubules, with findings such as tubular dilatation, epithelial cell flattening, and sloughing (Figure 1A). Large, degenerative nuclei were observed (Figure 1A(a,b)). Focal interstitial fibrosis and inflammatory cell infiltration (lymphocytes, neutrophils, and plasma cells) were present. Immunofluorescence showed no deposition of immunoglobulins or complement. Immunohistochemistry revealed sloughing of CD10-positive proximal tubular epithelial cells with tubular dilatation, while CK7-positive distal tubules maintained nuclear structure. Inflammatory cells consisted mainly of CD3-, CD4-, and CD8-positive T-cells, with no CD20-positive B-cells observed. Scattered CD68-positive macrophages and Granzyme B-positive cells were also noted (Figure 1B). Electron microscopy showed mild collapse of glomerular capillary loops, partial foot process effacement, increased mesangial matrix, and expansion of the subendothelial space (Figure 1C, upper panel). Mottled lysosomes were scattered within tubular cells (Figure 1C, lower panel). On day 12, treatment with prednisone was initiated at 30 mg/day. After steroid therapy, improvements were observed in urinary biomarkers including NAG index, serum uric acid, serum potassium, and serum phosphate levels. Nevertheless, on day 86, serum Cr level remained elevated at 1.41 mg/dL, indicating ongoing kidney dysfunction (Figure 2A,B). 

### 2.2. Case 2

**Figure 3:**
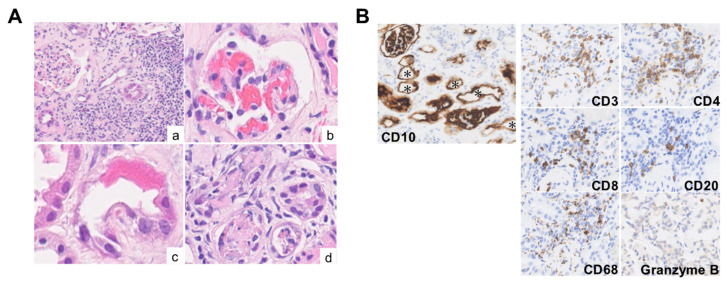
Kidney biopsy sample in Case 2. (**A**) Hematoxylin and eosin staining, magnification ×400. Tubular injury, mainly in the proximal tubules, is observed as a dilated tubular lumen and flattening and loss of tubular epithelium. Degenerated large nuclei (**a**,**c**), glomerular congestion (**b**), and epithelial cell shedding (**d**) are evident. (**B**) Immunohistochemistry, magnification ×400. Tubular epithelial desquamation and tubular dilatation (*) are seen in the CD10-positive proximal tubules. Infiltrating inflammatory cells are predominantly T cells, although the appearance of CD20-positive cells is also observed. Granzyme B-positive cells are slightly more prominent than in Case 1.

**Figure 4:**
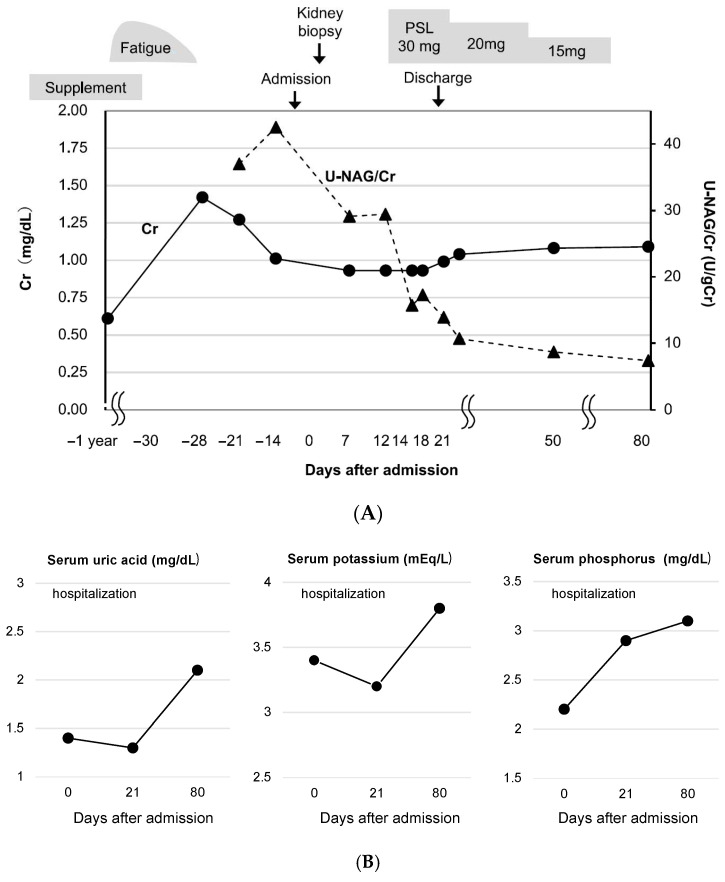
Clinical course in Case 2. (**A**) Clinical course. (**B**) Changes in serum levels of uric acid, potassium and phosphorus.

A 54-year-old woman had been self-administering red yeast rice supplements for approximately three years to manage dyslipidemia, despite not being prescribed any pharmacological treatment. She had no significant medical history. During a health check-up six months prior to hospitalization, serum Cr level was 0.61 mg/dL, with no urinary abnormalities detected. She noticed foamy urine about six weeks before admission and discontinued the red yeast rice supplements one month before admission. Shortly thereafter, she developed generalized fatigue and visited a local clinic, where deteriorating renal function (serum Cr, 1.42 mg/dL) and abnormal urinalysis findings (urinary protein 2+, urinary occult blood 2+, urinary glucose 2+) were noted. She was subsequently admitted to our department. On admission, the patient’s height and weight were recorded as 156.2 cm and 48.4 kg, respectively. Physical examination revealed a body temperature was 37.0 °C, blood pressure was 159/76 mmHg, and heart rate was 100 beats/min. Physical examination revealed no rash, joint pain, or other notable findings. Laboratory tests indicated renal dysfunction, with serum Cr at 1.27 mg/dL and an eGFR of 34.6 mL/min/1.73 m^2^. Urinalysis showed significant proteinuria (urinary protein, 1.46 g/g Cr) and elevated markers of tubular injury, including urinary NAG (34.6 U/L) and β2-MG (56,065 μg/L). Additional findings included hypouricemia (uric acid, 1.3 mg/dL), hypokalemia (potassium, 3.2 mEq/L), and hypophosphatemia (phosphate, 1.5 mg/dL), along with reduced tubular reabsorption of phosphate (%TRP, 53.0%). Severe glycosuria (urinary glucose 4+) and features of Fanconi syndrome were observed. Abdominal CT showed no renal atrophy. Kidney biopsy performed on hospital day 2 revealed segmental sclerosis in 5 of 24 glomeruli under light microscopy (Figure 3). Similar to Case 1, tubular findings included glomerular congestion (Figure 3A(b)), nuclear disappearance and enlarged degenerated nuclei (Figure 3A(c)), and epithelial cell shedding (Figure 3A(d)). Interstitial infiltration of lymphocytic inflammatory cells was also present. Fluorescence immunostaining showed no deposition of immunoglobulins or complement, and immunohistochemical staining revealed dilation of proximal tubular lumens positive for CD10. Interstitial inflammation predominantly comprised T cells (Figure 3B). Treatment with prednisolone was initiated at 30 mg/day on hospital day 8. Following discontinuation of the red yeast rice supplements, improvements were observed in urinary NAG levels and serum levels of uric acid, potassium, and phosphorus. Renal function also showed a gradual improvement, with serum Cr levels decreasing to 1.09 mg/dL by day 80 of hospitalization, but normal renal function had not yet been restored (Figure 4A,B).

### 2.3. Evaluation of AKI Markers

**Figure 5:**
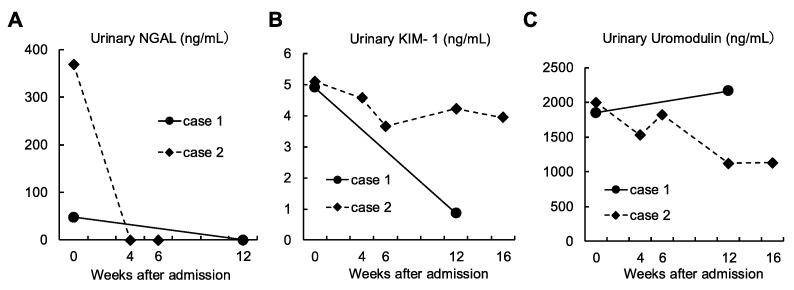
Urinary biomarkers changes in urinary levels of NGAL (**A**), KIM-1 (**B**) and uromodulin (**C**) are shown.

Urinary Neutrophil Gelatinase-Associated Lipocalin (NGAL), urinary Kidney Injury Molecule-1 (KIM-1), and urinary uromodulin were measured in both cases (Figure 5). These markers were measured by enzyme-linked immunosorbent assay (ELISA). Urinary NGAL was elevated in both cases before treatment, but decreased below the threshold of detection after treatment (Figure 5A). Urinary KIM-1 (Figure 5B) and urinary uromodulin (Figure 5C) recovered after starting treatment in Case 1, whereas, no recovery was observed in Case 2, suggesting prolonged tubular injury.

### 2.4. Renal Transporter Expression by Immunostaining

**Figure 6:**
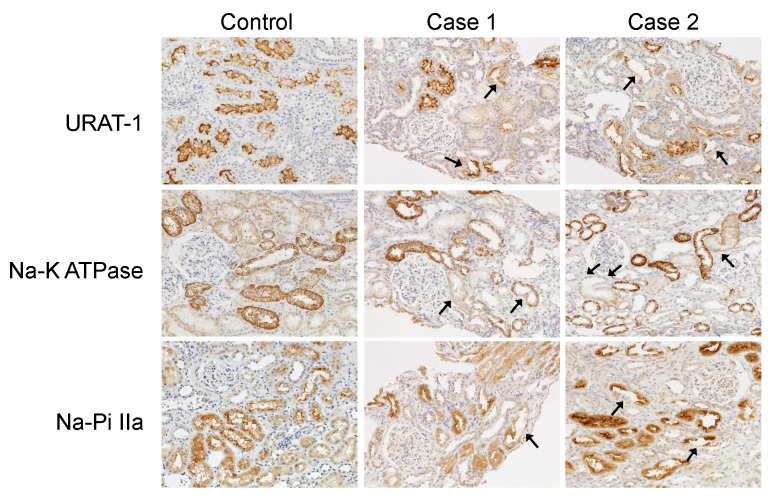
Expression of renal transporters in the kidney. URAT-1, Na-K ATPase, and Na-Pi IIa are evaluated by immunostaining. Magnification ×200.

Expressions of renal transporters including URAT-1, Na-K ATPase, and Na-Pi IIa in kidney biopsy specimens from Cases 1 and 2 were assessed by immunostaining. All three transporters were expressed in the tubules of normal kidneys obtained from non-tumorous portions of human renal cancer specimens without renal dysfunction. However, in both of our cases, a decrease was seen in the staining intensity of these transporters in some tubular segments (arrows) (Figure 6).

## 3. Discussion

Fanconi syndrome is characterized by a generalized dysfunction of the proximal renal tubules, manifesting as glucosuria, phosphaturia, generalized aminoaciduria, and type II renal tubular acidosis. Additional clinical features may include hypokalemia, sodium depletion, and dehydration. In pediatric populations, Fanconi syndrome is predominantly attributed to congenital metabolic disorders, however, in adults, its etiology primarily involves exposure to certain pharmacological agents, exogenous toxins, and heavy metals [9]. Although drug-induced Fanconi syndrome is uncommon, it arises from proximal tubular injury associated with the administration of alkylating agents, platinum-based compounds, nucleotide reverse transcriptase inhibitors, anticonvulsants, and aminoglycoside antibiotics. The abundance of organic acid transporters on the membranes of proximal tubular cells facilitates drug accumulation within these cells. Electron microscopic examination in cases of drug-induced Fanconi syndrome often reveals cellular swelling and aberrant mitochondrial morphology in proximal tubular epithelial cells. These findings support the hypothesis that Fanconi syndrome results from mitochondrial toxicity, leading to impaired ATP production, alongside disruption of receptor-mediated endocytosis via megalin and cubilin. Discontinuation of the offending agent typically results in partial or full recovery of tubular function, however, persistent tubular injury has been documented in certain cases [10].

Renal damage associated with red yeast rice supplements has been documented in 17 case reports involving 28 individual patients to date (Table 1) [11,12,13,14,15,16,17,18,19,20,21,22,23,24,25,26,27]. In the majority of cases, the primary clinical manifestations observed were Fanconi syndrome and metabolic acidosis. Proximal tubular cells were primarily affected in most instances. Conversely, tubulointerstitial nephritis was not detected in certain cases (Table 2). Corticosteroid therapy was administered to 12 out of 30 patients, however, renal dysfunction persisted in a significant proportion of these cases (Table 3).

Both of our cases exhibited mild tubulointerstitial nephritis, characterized by the infiltration of T lymphocytes into the interstitial space. This finding suggests a potential pathogenic role of T cell infiltration in immune-mediated interstitial nephritis. In both cases, corticosteroid therapy was initiated in anticipation of therapeutic benefit, given the presence of mild tubulointerstitial nephritis, the limited reports at the time linking red yeast rice supplementation to renal injury, and the absence of a definitive conclusion regarding the underlying etiology. On the other hand, the presence of severe tubular epithelial detachment suggested direct damage to the tubules by the causative substance. Supporting data included elevated levels of urinary NGAL and urinary KIM-1, which are biomarkers for AKI. In addition, urinary uromodulin, which reflects the proportion of normal tubules [28], was decreased. Considering the sustained elevation of AKI biomarkers at 1 month after treatment, puberulic acid may accumulate in tubular cells, contributing to prolonged declines in renal function.

Reduced expression of transporters in the proximal tubules has been reported in acquired Fanconi syndrome [29]. Consistently, our analysis demonstrated partial downregulation of URAT-1, Na-K ATPase, and Na-Pi IIa in specific proximal tubules within the renal biopsy specimens from both cases, while expression of these transporters was preserved in other tubular segments. Given that URAT-1, Na-K ATPase, and Na-Pi IIa are not exclusively localized to proximal tubules and thus lack absolute specificity, and considering the limited sample size of only two cases, it is not possible to definitively conclude that transporter downregulation was restricted to proximal tubules. Nevertheless, our findings suggest that reduced transporter expression in compromised proximal tubules may contribute to the pathogenesis of Fanconi syndrome. This syndrome is hypothesized to result from transporter dysfunction, including impaired reabsorption of various electrolytes, secondary to intracellular accumulation of the causative agent within proximal tubular cells. Further validation using animal models and additional studies are warranted to elucidate the precise underlying mechanisms.

In conclusion, we presented two cases of renal injury associated with the consumption of Red Yeast Rice Choleste-help supplements. These findings suggest that red yeast rice supplements may lead to renal injury, primarily through direct proximal tubular necrosis induced by a nephrotoxic compound. Further studies are needed to clarify the precise mechanisms responsible for this form of kidney injury.

## Figures and Tables

**Table 1:** Summary of clinical symptoms of Fanconi syndrome.

Case	Age	Sex	Fanconi Syndrome	K (mEq/L)	FEK (%)	P (mg/dL)	FEP (%)	UA (mg/dL)	FEUA (%)	Urine Glucose	Metabolic Acidosis	HCO_3_^-^ (mEq/L)	Reference No.
1	49	F	+	2.2	19.9	1.2	27.2	1.4	51.2	4+	+	16.7	[12]
2	68	F	+	2.4	64.2	2.4	unknown	2.4	unknown	3+	+	13.6	[12]
3	74	M	-	4.1	unknown	unknown	unknown	7.9	unknown	-	-	unknown	[13]
4	56	F	+	3.2	16.9	1.4	59.2	1.4	68.7	3+	+	15.6	[14]
5	58	F	+	4.3	unknown	2.9	unknown	1.7	unknown	3+	-	unknown	[15]
6	59	F	+	2.5	unknown	3.3	(%TRP) 61.7	1.9	45.3	4+	+	14.3	[16]
7	48	M	-	3.9	unknown	2.7	unknown	6.6	unknown	-	-	unknown	[16]
8	47	F	+	4.1	unknown	1.6	(%TRP) 73.8	3.1	23.7	1+	-	unknown	[16]
9	47	F	+	3.6	unknown	3.7	unknown	2.5	unknown	4+	-	unknown	[17]
10	62	M	+	2.8	10.8	1.1	55.3	1	51.1	4+	-	unknown	[18]
11	66	F	+	3.6	unknown	1	unknown	1.4	unknown	3+	unknown	unknown	[19]
12	54	M	+	3.3	unknown	3.1	unknown	2.8	unknown	-	unknown	unknown	[19]
13	52	F	+	3.3	unknown	2	unknown	1.7	unknown	4+	+	18.9	[20]
14	51	F	+	2.7	unknown	2.6	unknown	2.2	unknown	3+	+	17.9	[21]
15	49	F	+	3.3	unknown	3	unknown	8.1	unknown	3+	+	13.5	[22]
16	55	M	+	2.5	unknown	2	unknown	1.2	unknown	4+	+	15.5	[22]
17	60	F	+	1.9	unknown	3	unknown	1.8	unknown	4+	+	10.5	[22]
18	51	F	+	4.1	unknown	1.6	73.8	3.1	23.7	1+	-	unknown	[23]
19	50	M	+	2.8	48.8	2.4	(%TRP) 20.4	1.8	84	4+	+	9.6	[24]
20	56	F	+	3.5	unknown	1.9	unknown	1.9	unknown	4+	+	20.4	[25]
21	56	M	unknown	unknown	unknown	unknown	unknown	unknown	unknown	4+	+	unknown	[25]
22	73	F	+	3.2	unknown	1.4	unknown	1.6	unknown	unknown	+	15.4	[26]
23	53	F	+	3.4	unknown	1.7	unknown	1.2	unknown	unknown	+	15.7	[26]
24	55	F	-	4.2	unknown	3.8	unknown	3.5	unknown	unknown	-	unknown	[26]
25	42	M	+	2.9	24.9	2	unknown	1.2	83.4	4+	+	16.8	[27]
26	83	F	unknown	2.7	unknown	3.3	unknown	unknown	unknown	unknown	unknown	unknown	[27]
27	43	M	+	3.4	17.5	1.8	(Tmp/GFP) 0.87 mg/dL	1.7	42	3+	+	18.1	[28]
28	57	F	+	3.7	13.7	1.7	(Tmp/GFP) 0.87 mg/dL	1.6	43.4	4+	+	20.3	[28]
Our Case 1	44	M	+	3.5	14	2.7	(%TRP) 74.6	3.4	22.3	3+	-	24.2	
Our Case 2	57	F	+	3.2	17.8	1.5	(%TRP) 53.0	1.3	58.4	4+	+	22.1	

**Table 2:** Summary of histopathological findings.

Case	Glomerular Injury	Acute Tubular Necrosis	Tubular Atrophy	Tubulointerstitial Cell Infiltration	Immunostaining	Reference No.
1	Minor glomerular abnormality	+	+	-	Megalin was expressed in the injured tubular epithelium.	[12]
2	Minor glomerular abnormality	+	+	-	Megalin was expressed in the injured tubular epithelium.	[12]
3	Membranous nephropathy	+	+	-	not performed	[13]
4	Minor glomerular abnormality	+	+	±	not performed	[14]
5	Minor glomerular abnormality	+	+	Lymphocyte and plasmacyte	not performed	[15]
6	Minor glomerular abnormality	+	+	+	not performed	[16]
7	unknown	unknown	unknown	unknown	not performed	[16]
8	unknown	unknown	unknown	unknown	not performed	[16]
9	Some glomeruli showed global sclerosis, while others showed no abnormality.	+	-	-	not performed	[17]
10	Minor glomerular abnormality	+	-	-	not performed	[18]
11	Minor glomerular abnormality	+	-	-	not performed	[19]
12	Minor glomerular abnormality	+	-	Inflammatory cells	not performed	[19]
13	Minor glomerular abnormality	+	-	Mild lymphocytic infiltration	not performed	[20]
14	Minor glomerular abnormality	+	+	±	not performed	[21]
15	Minor glomerular abnormality	+	+	Mainly CD3+ T lymphocytes	not performed	[22]
16	unknown	unknown	unknown	unknown	not performed	[22]
17	unknown	unknown	unknown	unknown	not performed	[22]
18	Minor glomerular abnormality	+	+	-	SGLT2 expression was reduced in KIM-1 positive injured tubular cells. SGLT1 or GLUT2 staining showed no remarkable change.	[23]
19	Minor glomerular abnormality	+	+	-	not performed	[24]
20	Minor glomerular abnormality	+	+	insignificant	not performed	[25]
21	No abnormalities except diabetic nephropathy	+	+	insignificant	not performed	[25]
22	Minor glomerular abnormality	+	+	+	not performed	[26]
23	Minor glomerular abnormality	+	+	Severe inflammatory cell infiltration	not performed	[26]
24	Minor glomerular abnormality	+	+	Severe inflammatory cell infiltration	not performed	[26]
25	Minor glomerular abnormality	+	+	-	not performed	[27]
26	unknown	unknown	unknown	unknown	not performed	[27]
27	Minor glomerular abnormality	+	+	Mild mononuclear cell infiltration	NCC positive cells occasionally desquamated.	[28]
28	Minor glomerular abnormality	+	+	Focal inflammatory cell infiltration	NCC were preserved.	[28]
Our Case 1	Mild mesangial matrix expansion	+	-	Focal inflammatory cell infiltration	Decreased staining of URAT-1, Na-K ATPase, and Na-Pi II was observed in some tubular segments.	
Our Case 2	Some glomeruli showed segmental sclerosis, while others showed no abnormality.	+	-	Focal inflammatory cell infiltration	Decreased staining of URAT-1, Na-K ATPase, and Na-Pi II was observed in some tubular segments.	

**Table 3:** Summary of treatment and renal function.

Case	Treatment	Serum Cr Level (mg/dL)		Reference No.
		Before Treatment	After Treatment	
1	-	1.5	0.74	[12]
2	-	3	1.34	[12]
3	PSL 40 mg	1.21	0.98	[13]
4	PSL 30 mg	1.39	0.93	[14]
5	PSL 20 mg	1.57	unknown	[15]
6	-	2.32	0.96	[16]
7	-	0.87	0.92	[16]
8	-	1.05	1.13	[16]
9	PSL 40 mg	4.7	1.72	[17]
10	-	1.43	1.21	[18]
11	-	1.74	1.09	[19]
12	-	1.31	unknown	[19]
13	-	2.28	0.73	[20]
14	-	3.18	1.59	[21]
15	PSL 50 mg	21.9	1.66	[22]
16	-	2.33	1.08	[22]
17	-	3.71	1.11	[22]
18	PSL 30 mg→discontinued five days after initiation	1.1	0.79	[23]
19	-	3.99	1.88	[24]
20	-	1.89	1.04	[25]
21	PSL 30 mg	13.55	1.69	[25]
22	PSL (dosage was not mentioned)	1.27	unknown	[26]
23	Steroid pulse and PSL (dosage was not mentioned)	1.41	unknown	[26]
24	Steroid pulse and PSL (dosage was not mentioned)	2.38	unknown	[26]
25	mPSL 500 mg for 3 days, followed by PSL 30 mg	2.12	1.03	[27]
26	-	3.52	Not recovered	[27]
27	-	1.96	0.9	[28]
28	-	1.75	1.3	[28]
Our Case 1	PSL 30 mg	1.58	1.41	
Our Case 2	PSL 30 mg	1.27	1.09	

FEK: fractional excretion of Potassium, FEP: fractional excretion of Phosphate, %TRP: percentage of tubular reabsorption of Phosphate, FEUA: fractional excretion of Uric Acid, ATN: acute tubular necrosis, mPSL: methylprednisolone.

## Data Availability

The original contributions presented in this study are included in the article. Further inquiries can be directed to the corresponding author.
